# The relationship between lifestyle behaviors and tension-type headache and migraine: A systematic review and meta-analysis

**DOI:** 10.1097/MD.0000000000046868

**Published:** 2025-12-26

**Authors:** Tao Gao, Hongdan Luo, Xiyan Zhang, Xiaxia Jin, Zhengquan Lei

**Affiliations:** a College of Acupuncture and Massage, Shaanxi University of Chinese Medicine, Xianyang, China; b Department of Neurology, Baoji People’s Hospital, Baoji, China; c Basic Medical College, Shaanxi University of Chinese Medicine, Xianyang, China; d National Resource Center for Chinese Materia Medica, China Academy of Chinese Medical Sciences, Beijing, China; e Department of Acupuncture and Tuina, The Affiliated Hospital of Shaanxi University of Chinese Medicine, Xianyang, China.

**Keywords:** lifestyle behaviors, meta-analysis, migraine, systematic review, tension-type headache

## Abstract

**Background:**

Despite the growing recognition of lifestyle factors as potential modifiable influencers of headache disorders, research on the association between lifestyle behaviors and migraine or tension-type headache (TTH) remains limited. Therefore, the objective of this study was to evaluate the potential association between lifestyle behaviors and these 2 headache disorders (migraine and TTH).

**Methods:**

A comprehensive database search was conducted in PubMed, Embase, Cochrane Library, Scopus, and Web of Science up to May 2024 to identify observational studies. Relative risk (RR) values were extracted from the eligible studies, and pooled RRs were calculated through meta-analysis. Two independent investigators performed data screening to ensure the accuracy and reliability of the data.

**Results:**

A total of 7 studies (32,197 participants) were included. The results showed no significant association between alcohol consumption and migraine (RR = 0.87, 95% confidence interval (CI): 0.47–1.62), body mass index (BMI) and migraine (RR = 1.13, 95% CI: 0.87–1.48), or smoking/physical activity/alcohol consumption and TTH (RR = 1.12, 95% CI: 0.96–1.30; RR = 0.91, 95% CI: 0.70–1.20; RR = 1.07, 95% CI: 0.78–1.45, respectively).

**Conclusion:**

Consistent with these findings, the present study concludes that no significant association was observed between lifestyle behaviors (alcohol consumption, BMI, smoking, physical activity) and the risk of migraine or TTH. Further studies are warranted to explore this association. However, only 7 studies were included in this analysis, so the conclusion requires further verification through additional high-quality research.

## 1. Introduction

Migraine is a highly prevalent and disabling neurological disorder that manifests as recurrent headache attacks, often accompanied by nausea and hypersensitivity to light and sound (photophobia and phonophobia).^[1]^ Migraine affects approximately 12% of the global population and represents the leading neurological cause of years lived with disability. In roughly 20% of individuals with migraine, sensory alterations-predominantly visual disturbances-occur immediately prior to headache onset; this phenomenon is termed an aura.^[2,3]^ Tension-type headache (TTH) is a prevalent primary headache in clinical practice, primarily distinguished by non-pulsating, bilateral pain of mild to moderate intensity that presents as a sensation of tightness or pressure. According to the Global Burden of Disease Study 2023, approximately 52.0% of the global population experiences headaches, with TTH accounting for 26.0% of these cases.^[4]^ The annual prevalence of frequent episodic TTH is approximately 21.6%^[5]^; TTH patients constitute 60% of the headache population, with reports indicating that pain impairs their daily activities, work productivity, and social functioning.^[6,7]^

Lifestyle modifications represent a cornerstone in the management of migraine and TTH. Key recommendations include maintaining circadian regularity via consistent sleep-wake cycles, adhering to a balanced diet, engaging in moderate aerobic exercise (3–4 sessions per week, 30 minutes per session), and practicing stress-reduction techniques (such as mindfulness meditation). These interventions have been proven effective in randomized controlled trials to reduce monthly migraine frequency by 30 to 40% and alleviate headache-related functional impairment. Clinical studies have also confirmed the individual efficacy of each component, thereby reinforcing their role as evidence-based interventions.^[8–12]^

Migraine and TTH impose significant burdens on individuals, society, and the economy. While lifestyle factors (e.g., diet, sleep, physical activity) are widely recognized as modifiable influencers of chronic pain conditions, critical evidence gaps persist: most prior studies on lifestyle-headache associations are small-scale, cross-sectional, or focus solely on migraine, with inconsistent findings on TTH and limited synthesis of data across populations. Addressing this gap is urgent, as targeted lifestyle interventions could serve as low-cost, accessible strategies to mitigate headache burden – yet their effectiveness depends on a clear understanding of which lifestyle factors are truly associated with migraine/TTH risk.

This study hypothesized that specific lifestyle behaviors (e.g., alcohol consumption, irregular sleep, sedentary behavior) are significantly associated with the risk of migraine and TTH, with consistent directional effects across eligible observational studies. Unlike previous reviews that focused exclusively on migraine or omitted key lifestyle factors (e.g., circadian rhythm disruptions), this study conducts a comprehensive systematic review and meta-analysis of observational studies, simultaneously evaluating lifestyle associations with both migraine and TTH. By pooling data from diverse populations and standardizing effect sizes (e.g., relative risk [RR]), this work provides the first integrated evidence base to guide clinical recommendations for lifestyle-based headache prevention.

The wider impact of this study is threefold: for clinicians, it offers evidence-based guidance to tailor lifestyle counseling for patients with migraine/TTH; for public health policymakers, it identifies prioritized lifestyle targets (e.g., improving sleep regularity) to reduce population-level headache burden; and for researchers, it highlights unresolved gaps (e.g., dose-response relationships between lifestyle factors and headaches) to inform future longitudinal studies. To elucidate these associations and address existing evidence limitations, this study conducted a systematic review and meta-analysis of observational studies.

## 2. Materials and methods

This meta-analysis adhered to the Preferred Reporting Items for Systematic Reviews and Meta-analyses (2020) guidelines for reporting. Its registration number is CRD420251052624.

### 2.1. Methods

To identify studies on lifestyle behaviors in relation to tension-type headache (TTH) and migraine, we conducted a systematic search of PubMed, Embase, the Cochrane Library, Scopus, and Web of Science up to May 15, 2025. The search was performed using the terms (“lifestyle factors” OR “behavior” OR “healthy lifestyle” OR “lifestyle modification” OR “healthy habits” OR “healthy living”) AND (“tension - type headache” OR “tension headache” OR “migraine”). Two researchers independently screened the studies. Discrepancies were resolved by a third researcher through full-text review. We imposed no restrictions on language or filters. Furthermore, to ensure comprehensiveness, we hand – searched the reference lists of relevant reviews, articles, and books to identify additional references.

### 2.2. Inclusion and exclusion criteria

The meta-analysis included studies that met the following criteria: Observational designs (case control, cohort, or cross-sectional); Assessment of lifestyle factors, including smoking status, alcohol consumption, dietary patterns, physical activity, sleep behaviors, body weight, and mental activity; and Studies reporting effect estimates with 95% confidence intervals (CIs) or providing data to calculate RRs for the association between lifestyle factors and the incidence of TTH or migraine.

Studies were excluded based on the following criteria: publications available only as letters, abstracts, or conference proceedings; failure to report quantitative outcome data; non-English language; and inaccessible full text despite reasonable attempts to obtain it.

### 2.3. Study selection and data extract

Before the review process, a pre-designed Excel spreadsheet was used to extract data from the selected studies. Two researchers collected the following information: general details (e.g., title, author, study country, year of publication), study characteristics (e.g., design), study population (e.g., age, sex, sample size), exposure characteristics (e.g., type of headache, follow-up duration), and outcomes (e.g., primary and secondary outcomes, assessment time points, evaluation methods). When necessary, original study authors were contacted to request additional data or clarifications. Any discrepancies in data extraction were resolved through discussion until a consensus was reached.

### 2.4. Study quality assessment

The quality of the included cohort studies was assessed using the Newcastle–Ottawa scale (NOS), which evaluates 3 domains: study group selection, comparability of groups, and outcome assessment. A NOS score of 7 or higher is considered indicative of high quality, whereas lower scores suggest a higher risk of bias. For cross-sectional studies, quality assessment was performed using the 2013 version of the Agency for Healthcare Research and Quality criteria. This tool consists of 11 items, with responses categorized as “yes,” “no,” or “unclear,” where each “yes” receives 1 point and “no” or “unclear” responses receive 0 points. The total score (0–11) is categorized as: low quality (0–3), moderate quality (4–7), and high quality (8–11). Study quality was initially assessed by one author, and the evaluations were independently verified by a second author. Any discrepancies were resolved through discussion and consensus.

### 2.5. Statistical analysis

RRs were utilized as the unified effect metrics to quantify the strength of associations between lifestyle behaviors and incident cases of TTH or migraine. Given the low incidence rates, odds ratios from primary studies were considered approximations of RRs. A random-effects model was employed, using the group with minimal lifestyle modifications as the reference category. Heterogeneity across studies was assessed using Cochran *Q* statistic and the *I*^2^ statistic. An *I*^2^ value exceeding 50% was considered indicative of significant inter-study heterogeneity.

For meta-analyses involving ≥10 studies, publication bias was evaluated through funnel plot analysis and Egger test for asymmetry. In cases where significant publication bias was detected, the trim- and -fill method was applied to evaluate the impacts of missing studies on the pooled effect size.

## 3. Results

### 3.1. Study characteristic

**Figure 1.**
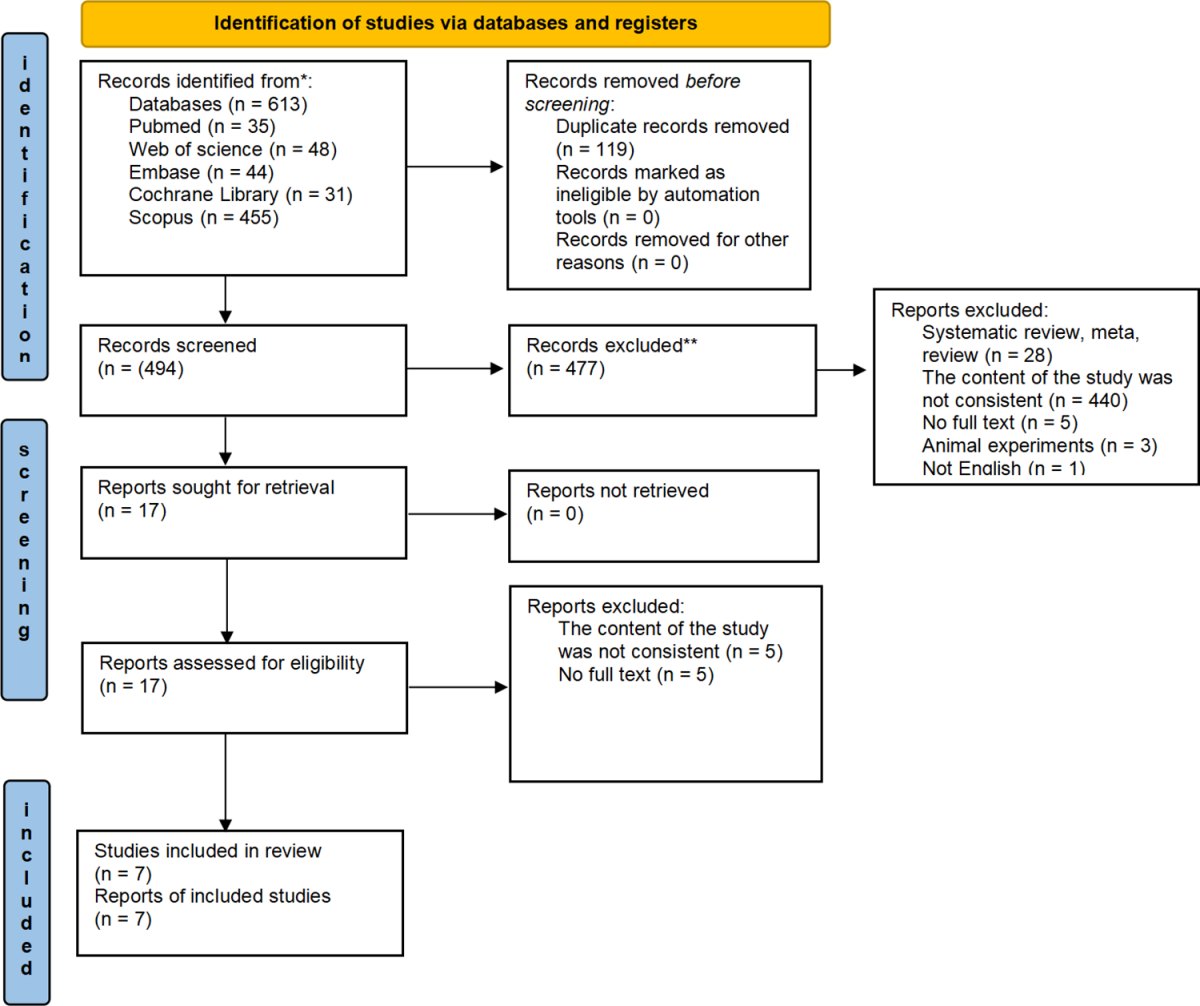
Flow diagram of the study selection process.

A systematic search was conducted to identify eligible studies for this meta-analysis, as depicted in Figure 1. The search initially yielded 613 studies, which were subjected to a comprehensive review process: duplicate removal, title or abstract screening, and full-text evaluation. Ultimately, 606 articles were excluded for being literature reviews, systematic reviews, animal studies, or conference abstracts. A total of 7 articles^[13–19]^ were included in this study.

The total sample size ranged from 644 to 13,498 participants, with a median of approximately 3000. The proportion of males varied widely, from 0%^[19]^ to 48.25%,^[17]^ with most studies showing a male ratio <30%. The age span was 14 to >80 years, with adults (≥18 years) as the primary study population. Cohort studies^[13–15,18]^ accounted for 50% (n = 4) as the main design type, followed by one longitudinal study,^[17]^ one cross-sectional survey,^[19]^ and one database-based cohort study.^[16]^ Only one study^[15]^ simultaneously compared migraine with TTH.

Exposure factors focused on: Lifestyle: body mass index (BMI), smoking, alcohol consumption, physical activity, sleep patterns; Occupational and environmental: shift work, job stress, and physical work burden; Psychosocial: family dysfunction, parenting style, and perceived stress.

For outcome assessment, 5 studies used self-reported questionnaires (such as International Headache Society criteria, ICD-10 coding),^[13,14,16,17,19]^ 1 relied on clinical diagnosis (Nord-Trøndelag Health Study 3 database),^[15]^ and 1 used physician-confirmed reports^[18]^ (see Table 1 for details).

**Table 1:** Characteristics of included studies.

Study	Country	Design	Sample size	Males (%)	Mean or median age (yr)	Occupation	Follow-up years	Outcome, assessment	Lifestyle composition
Migraine									
Appel AM^[14]^ (2020)	Denmark	Cohort	2272	27.8 vs 14.7	47.0 (9.7) vs 41.7 (10.0)	Various	/	Common mental disorder questionnaire	Shift workers, smoking, alcohol drinking, physical activity, body mass index, perceived stress, disturbed sleep, difficulties awaking
Evans EW^[19]^ (2015)	USA	Cross-sectional surveys designed	3023	0	35.5 ± 0.4 vs 35.6 ± 0.3	Various	/	Self-report in the NHANES pain questionnaire	Smoking, body mass index, alcohol drinking, dietary intake pattern
Le H^[17]^ (2013)	Denmark	Longitudinal study	13,498	48.25	18–41 yr	Various	/	Questionnaire	Environmental factors, hard physical work load, hard physical activity, BMI, alcohol consumption
Santos IS^[16]^ (2014)	Brazil	Brazilian longitudinal study of adult health (ELSA-Brasil).	6372	2116 (68.0) vs 770 (23.6)	52 (46, 47) vs 48 (43, 52)	Various	12 mo	A detailed questionnaire based on the International Headache Society (IHS) criteria	Job stress
Hagen K^[15]^ (2018)	Norway	Cohort	644	243 (37.73)	42.8 (14.9)	Various	/	Headache diagnosis in HUNT3	Smoking, alcohol drinking, hard physical exercise, body mass index
Hammond NG^[18]^ (2019)	Canada	Cohort	2313	3.0	14–25 yr	Various	14 yr	Self-reported physician-diagnosed migraine	High family dysfunction, high hostile parenting, high punitive parenting, parental depressive symptoms, low symptoms, moderate symptoms, severe symptoms
Lei Y^[13]^ (2024)	China	UK Biobank cohort study	3225	913 (28.3)	58.00 yr	Various	13.58 yr	Tenth revision of the International Classification of Diseases (ICD-10)	BMI, smoking status, alcohol consumption, physical activity, diet, sleep pattern and sedentary time
TTH									
Hagen K^[15]^ (2018)	Norway	Cohort	850	404 (47.53)	41.9 (12.6)	Various	/	Headache diagnosis in HUNT-3	Smoking, alcohol drinking, hard physical exercise, body mass index

BMI = body mass index, HUNT3 = Nord-Trøndelag Health Study 3, NHANES = National Health and Nutrition Examination Surveys, TTH = tension-type headache.

### 3.2. Quality assessment of included literature

All 7 studies were deemed to be of high quality, with quality assessment scores ranging from 7 to 9. Cohort studies achieved an average NOS score of 8, demonstrating strength in selection, comparability, and follow-up domains. Conversely, cross-sectional studies obtained an average score of 8 on the Agency for Healthcare Research and Quality scale, meeting 8 out of 11 assessment items. No significant bias was identified in either the selection or assessment processes, thereby ensuring the reliability of the pooled results (see Tables 2 and 3 for details).

**Table 2:** Quality evaluation of included studies.

Study (cohort)	Representative of the exposed cohort	Selection of non-exposed cohort	Ascertainment of exposure	Outcome not present before study	Comparability	Assessment of outcome	Follow-up long enough	Adequacy of follow up	Quality score
Appel AM^[14]^ (2020)	✳	✳	✳	✳	✳✳	✳			7
Le H^[17]^ (2013)	✳	✳	✳	✳	✳✳	✳			7
Santos IS^[16]^ (2014)	✳	✳	✳	✳	✳✳	✳	✳	✳	9
Hagen K^[15]^ (2018)	✳	✳	✳	✳	✳✳	✳			7
Hammond NG^[18]^ (2019)	✳	✳	✳	✳	✳✳	✳	✳	✳	9
Lei Y^[13]^ (2024)	✳	✳	✳	✳	✳✳	✳	✳	✳	9

✳ Indicates a score of one.

**Table 3:** Quality evaluation of included studies.

List of items	Evans EW^[19]^ (2015)
1.Is the source of the data identified (survey, literature review)?	✳
2.Are the inclusion and exclusion criteria for the exposed and unexposed groups (cases and controls) listed?	✳
3.Is a period given for the identification of patients?	✳
4.Were the subjects continuous, if not population-derived?	✳
5.Do the subjective factors of the evaluators obscure other aspects of the subjects?	✳
6.The rationale for excluding any patients from the analysis was explained.	✳
7.Describe measures to evaluate and/or control for confounding factors.	✳
8.If possible, explain how missing data were handled in the analysis.	
9.The summary of patient response rates and the completeness of data collection.	✳
10.Response rates and the completeness of data collection are summarized.	
11.If there is a follow-up, identify the percentage of patients with expected incomplete data or follow-up results.	
Total quality assessment score	8

### 3.3. The relationship between alcohol consumption and migraine

**Figure 2.**
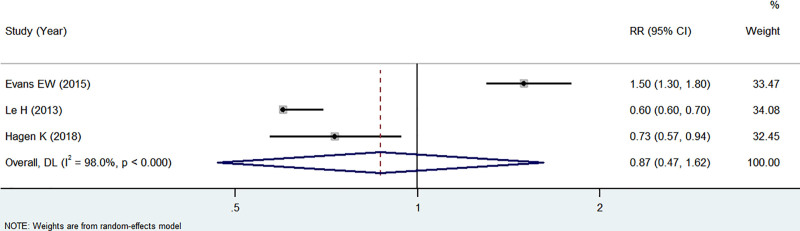
Forest plot of the relationship between alcohol consumption and migraine.

Three articles^[15,17,19]^ reported the association between alcohol consumption and migraine. Due to substantial heterogeneity (*I*^2^ = 98%, *P* < .1), a random-effects model was used. No association was found between alcohol use and risk of migraine (*Z* = −0.444, *P* = .657, RR: 0.87, 95% CI: 0.47–1.62) (see Fig. 2 for details). It should be noted that these findings require cautious interpretation due to the limited number of included studies.

### 3.4. Subgroup analysis

We conducted a subgroup analysis to explore the potential impact of specific study characteristics on the results. Subgroup analyses were performed based on regions (Europe vs USA) and research quality scores (7 vs 8 points) (see Table 4). When analyzing regional subgroups, the pooled results showed that country/region and research quality score significantly influenced migraine risk (*P* < .01). This discrepancy may be attributed to differences in demographic characteristics of study population (e.g., age, sex ratio), and alcohol consumption amount (e.g., daily vs weekly intake). Notably, these findings should be interpreted with caution, primarily due to 2 key limitations: first, the limited number of included studies constrains the statistical power and generalizability of the results; second, several subgroup analyses conducted in this study are purely exploratory in nature, lacking sufficient statistical rigor and consistent validation to draw definitive conclusions.

**Table 4:** The results of subgroup analysis.

Subgroup	N	*I*^2^ (%)	*P* (heterogeneity)	RRs	Pooled model	Statistically significant *P*-values
USA						
Evans EW^[19]^ (2015)	3023	0%	<.01	1.5 (1.3–1.8)	Random effects model	<.01
Europe						
Hagen K^[15]^ (2018)	644	53.6%	.142	0.73 (0.57–0.94)	Random effects model	<.01
Le H^[17]^ (2013)	13,498	53.6%	.142	0.6 (0.6–07)	Random effects model	<.01
7 score						
Hagen K^[15]^ (2018)	644	53.6%	.142	0.73 (0.57–0.94)	Random effects model	<.01
Le H^[17]^ (2013)	13,498	53.6%	.142	0.6 (0.6–07)	Random effects model	<.01
8 score						
Evans EW^[19]^ (2015)	3023	0%	<.01	1.5 (1.3–1.8)	Random effects model	<.01

RR = relative risk, USA = United States of America.

### 3.5. The relationship between BMI and migraine

**Figure 3.**
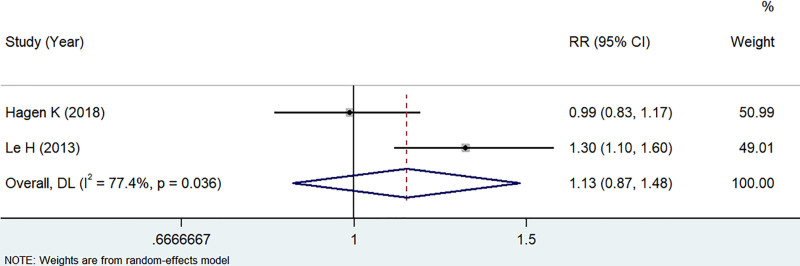
Forest plot of the relationship between BMI and migraine. BMI = body mass index.

Two articles^[15,17]^ reported the association between BMI and migraine. Due to substantial heterogeneity (*I*^2^ = 77.4%, *P* = .036), a random-effects model was used. No association was found between BMI and risk of Migraine (*Z* = 0.907, *P* = .365, RR: 1.13, 95% CI: 0.87–1.48) (see Fig. 3 for details). Due to the limited number of included studies, subgroup analysis was not performed. It should be noted that these findings require cautious interpretation due to the limited number of included studies.

#### 3.5.1. The relationship between smoking, physical activity, alcohol use and tension-type headache (TTH)

One article^[15]^ reported the association between smoking, physical activity, alcohol use and risk of TTH. No significant association was found between smoking, physical activity, alcohol consumption, and the risk of TTH (RR = 1.12, 95% CI: 0.96–1.30; RR = 0.91, 95% CI: 0.70–1.20; RR = 1.07, 95% CI: 0.78–1.45; respectively) (see Table 5 for details).

**Table 5:** The other influencing factors included in the study.

Influencing factors	Study	Year	RR/OR/HR (95% CI)
Migraine			
Alcohol consumption	Evans EW	2015	OR: 1.5, 95% CI: 1.3–1.8
	Hagen K	2018	RR: 0.73, 95% CI: 0.57–0.94
	Le H	2013	OR: 0.6, 95% CI: 0.6–0.7
BMI	Hagen K	2018	RR: 0.99, 95% CI: 0.83–1.17
	Le H	2013	OR: 1.3, 95% CI: 1.1–1.6
Hard physical work load	Le H	2013	OR: 1.1, 95% CI: 1.0–1.2
High job demands	Santos IS	2014	OR: 1.37, 95% CI: 1.18–1.59
Hard physical activity	Le H	2012	OR: 1.2, 95% CI: 1.0–1.3
Low job control	Santos IS	2014	OR: 1.30, 95% CI: 1.10–1.53
Low social support	Santos IS	2014	OR: 1.49, 95% CI: 1.29–1.71
Smoking	Hagen K	2018	RR: 1.30, 95% CI: 1.11–1.52
Physical exercise	Hagen K	2018	OR: 0.71, 95% CI: 0.54–0.94
High family dysfunction	Hammond NG	2019	OR: 0.84, 95% CI: 0.54–1.32
High Hostile parenting	Hammond NG	2019	OR: 1.28, 95% CI: 0.81–2.01
High Punitive parenting	Hammond NG	2019	OR: 1.27, 95% CI: 0.81–2.00
Parental depressive symptoms			
Low symptoms	Hammond NG	2019	OR: 0.83, 95% CI: 0.47–1.47
Moderate symptoms	Hammond NG	2019	OR: 0.85, 95% CI: 0.44–1.65
Severe symptoms	Hammond NG	2019	OR: 0.87, 95% CI: 0.33–2.29
Ideal category of Healthy lifestyle	Lei Y	2024	HR: 0.81, 95% CI: 0.73–0.91
Shift work	Appel AM	2020	OR: 1.72; 95% CI: 1.04–2.86
Diet	Evans EW	2015	RR: 1.00, 95% CI: 0.93–1.09
TTH			
Smoking	Hagen K	2018	RR: 1.12, 95% CI: 0.96–1.30
Physical exercise	Hagen K	2018	RR: 0.91, 95% CI: 0.70–1.20
Alcohol consumption	Hagen K	2018	RR: 1.07, 95% CI: 0.78–1.45

BMI = body mass index, HR = hazard ratio, CI = confidence interval, OR = odds ratio, RR = relative risk, TTH = tension-type headache.

### 3.6. Comprehensive analysis of other major influencing factors of migraine that were not combined

#### 3.6.1. Smoking

Hagen et al^[15]^ demonstrated that smoking significantly increases the risk of migraine (RR = 1.30, 95% CI: 1.11–1.52), indicating that smoking is a well-established risk factor. Notably, these findings are limited by their reliance on a single cross-sectional study, which highlights the need for longitudinal studies in diverse populations to validate and expand upon these results (see Table 5 for details).

#### 3.6.2. Physical activity and exercise

Hagen et al^[15]^ found that hard physical exercise reduces migraine risk (odds ratio (OR) = 0.71, 95% CI: 0.54–0.94). Conversely, Le et al^[17]^ showed that hard physical activity (OR = 1.2, 95% CI: 1.0–1.3) may increase the risk, suggesting that moderate exercise is beneficial while excessive activity is harmful (see Table 5 for details).

#### 3.6.3. Occupational stress

Santos et al^[16]^ showed that high job demands (OR = 1.37, 95% CI: 1.18–1.59), low job control (OR = 1.30, 95% CI: 1.10–1.53), and low social support (OR = 1.49, 95% CI: 1.29–1.71) were all significantly associated with migraine risk. This finding indicates that workplace stress is a key trigger for migraines (see Table 5 for details).

#### 3.6.4. Physical load

Le et al^[17]^ found that heavy physical workload (OR = 1.1, 95% CI: 1.0–1.2) is associated with migraine, potentially mediated by muscle tension or metabolic stress.

#### 3.6.5. Shift work

Appel et al^[14]^ first reported that shift workers had a higher incidence of migraine than non-shift workers (OR = 1.72; 95% CI: 1.04–2.86), with a significant interactive effect with sleep disorders (e.g., difficulty falling asleep).

#### 3.6.6. Family function and parenting style

Hammond et al^[18]^ found that hostile/punitive parenting styles were associated with a modest increase in migraine risk (OR = 1.28, 95% CI: 0.81–2.01; OR = 1.27, 95% CI: 0.81–2.00), though neither estimate reached statistical significance. By contrast, family dysfunction^[18]^ was not significantly associated with migraine risk (OR = 0.84, 95% CI: 0.54–1.32). This suggests that specific early family stressors (e.g., parenting styles) may be more relevant to migraine risk than general dysfunctional environments.

#### 3.6.7. Healthy lifestyle

Lei et al^[13]^ demonstrated that a healthy lifestyle – including a balanced diet (e.g., a diet high in fruits/vegetables and low in processed foods) and a regular sleep-wake schedule (7–8 hours/night with ≤1-hour variation) – was associated with a 19% reduced migraine risk in a prospective cohort study (hazard ratio = 0.81, 95% CI: 0.73–0.91, *P* < .01). This finding highlights the importance of incorporating these modifications into clinical preventive strategies.

#### 3.6.8. Parental depressive symptoms

Hammond et al^[18]^ found no significant association between parental depressive symptoms and offspring migraine risk (OR = 0.83–0.87) (see Table 5 for details).

Notably, these findings are limited by their reliance on a single study. This limitation underscores the need for longitudinal studies in diverse populations to validate and expand these results.

### 3.7. Sensitivity analysis and evaluation for publication bias

**Figure 4.**
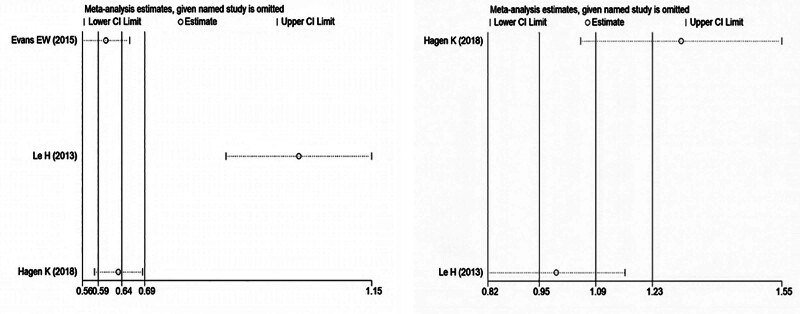
Sensitivity analysis of the relationship between alcohol consumption and BMI and migraine. BMI = body mass index.

Sensitivity analyses were performed by sequentially excluding each study one by one to assess the stability of pooled results, as shown in Figure 4. Egger test for publication bias showed no significant asymmetry (*P* = .563), indicating the robustness of the findings. However, due to the limited number of studies (n < 10), funnel plots were not generated.

## 4. Discussion

This review comprehensively retrieved all studies investigating the associations between Lifestyle Behaviors and TTH and Migraine. The 7 high-quality studies included in this article have a large combined sample size and can conduct reliable comprehensive analysis. To the best of our knowledge, this represents the meta-analysis focusing on the associations between lifestyle behaviors and the onset of migraine or TTH. Notably, both sensitivity analysis and publication bias assessments confirmed the robustness of the pooled estimates.

This meta-analysis included 7 studies with 32,197 participants. The analysis revealed no significant association between alcohol consumption and migraine risk (*Z* = −0.444, *P* = .657; RR = 0.87, 95% CI: 0.47–1.62), nor between BMI and migraine risk (*Z* = 0.907, *P *= .365; RR = 1.13, 95% CI: 0.87–1.48). For TTH, no significant associations were found between smoking (RR = 1.12, 95% CI: 0.96–1.30), physical activity (RR = 0.91, 95% CI: 0.70–1.20), or alcohol consumption and TTH risk (RR = 1.07, 95% CI: 0.78–1.45). We conducted a subgroup analysis to explore the potential impact of specific study characteristics on the results. Subgroup analysis were performed based on regions (Europe vs USA) and research quality scores (7 vs 8 points). When analyzing regional subgroups, the pooled results showed that country/region and research quality score significantly influenced migraine risk (*P* < .01). This discrepancy may be attributed to differences in study population, alcohol consumption amount, and definition of headache type. Notably, these findings should be interpreted with caution, primarily due to 2 key limitations: first, the limited number of included studies constrains the statistical power and generalizability of the results; second, several subgroup analyses conducted in this study are purely exploratory in nature, lacking sufficient statistical rigor and consistent validation to draw definitive conclusions.

Considering the complexity and diversity of lifestyle factors, these variables may confound research outcomes through interactions with genetic or environmental modifiers. The findings suggest that larger sample sizes and more sophisticated epidemiological approaches (e.g., genome-wide association studies combined with longitudinal lifestyle tracking) are required to elucidate the causal mechanisms underlying the association between lifestyle behaviors and migraine or TTH.

## 5. The underlying mechanisms of the results

The lack of significant association between alcohol consumption and migraine risk might be attributed to interindividual variations in genetic predisposition to neuroinflammation. Ethanol and congeners (e.g., histamine, serotonin) in alcoholic beverages are capable of activating transient receptor potential cation channel subfamily V member 1 and Toll-like receptor 4, thereby promoting the release of pro-inflammatory cytokines and triggering neuroinflammatory responses in the trigeminal vascular system.^[20]^ However, variations in congener composition across different alcoholic beverages – e.g., pro-inflammatory aldehydes in spirits versus anti-inflammatory flavonoids in red wine – may give rise to heterogeneous effects on headache risk. Additionally, the predominant consumption of red wine in European cohorts, which is rich in resveratrol, may mitigate headache risk by inhibiting nuclear factor kappa-B-mediated inflammation. In contrast, beer or spirit consumption in U.S. studies may lack such protective effects owing to their higher aldehyde content.^[20]^ Moreover, alcohol consumption in European populations is characterized by a higher frequency of intake but lower single-dose amounts, whereas U.S. populations tend to engage in binge-drinking patterns, which are more likely to precipitate headache episodes.^[21]^ Furthermore, genetic polymorphisms in alcohol-metabolizing enzymes have a significant impact on individual alcohol tolerance by modulating ethanol metabolism rates. Consequently, the insufficient incorporation of genotyping data in certain studies can result in selection bias, as individuals harboring specific enzyme polymorphisms may be disproportionately underrepresented.

The nonsignificant association between BMI and migraine risk may stem from the threshold effect of obesity-related metabolic abnormalities. Obesity contributes to migraine pathogenesis through inflammatory factors (e.g., interleukin-6, tumor necrosis factor-α) secreted by adipocytes and vasoactive substances (e.g., calcitonin gene-related peptide).^[22]^ However, BMI as a global marker of obesity cannot reflect visceral fat accumulation or specific phenotypes of metabolic syndrome. For example, BMI classification (e.g., overweight vs obesity) in certain studies may mask the specific impacts of visceral fat on neurovascular function. Moreover, the association between obesity and migraine may show sex differences: fat distribution in females is more likely to trigger estrogen fluctuations, while males may be susceptible to headache via androgen metabolic pathways. Nevertheless, existing studies have not sufficiently conducted sex-stratified analyses to explore these potential disparities.^[22]^

Furthermore, existing studies have not performed in-depth mechanistic analyses of headache subtypes, which may lead to effect difference. European studies more frequently employ the International Classification of Headache Disorders, 3rd edition criteria, while U.S. studies often depend on clinical diagnosis, giving rise to classification bias in headache types.

In summary, the associations between lifestyle factors and the risk of migraine or TTH are shaped by complex mechanisms spanning biological, psychological, and sociocultural dimensions. These factors are intricately interwoven and mutually reinforcing, contributing to the heterogeneity and complexity of research findings. Future investigations should further explore these mechanisms while prioritizing the control of confounding factors and enhancement of research quality, to more accurately clarify the relationship between lifestyle and headache risk. This will provide more robust evidence for preventive and therapeutic strategies in headache disorders.

## 6. Comparison with other studies

Although the meta-analysis included 32,197 participants, the sample sizes in subgroup analyses of Europe and the United States were each fewer than 10,000, which may limit the statistical power to detect small-effect associations. For instance, the threshold effect of alcohol intake might be obscured by inconsistencies in exposure measurement across studies. Additionally, variations in study quality scores (7 vs 8 points) could reflect differences in confounder control rigor, whereby more comprehensive covariate adjustment in high-quality studies might attenuate the observed associations of lifestyle factors.

A study^[23]^ conducted a systematic review and meta-analysis to explore the association between smoking and primary headaches (including migraine, TTH and cluster headache). The study found that current smoking was associated with an increased risk of migraine (OR = 1.29) and a decreased risk of TTH (OR = 0.78), but no direct causal relationship was established.

In contrast to previous studies that primarily focused on limited lifestyle factors (e.g., smoking or physical activity), our study incorporates a wider array of lifestyle behaviors – including Alcohol consumption, dietary patterns, BMI, Shift work, and stress management practices. This comprehensive inclusion allows for a more systematic analysis of the associations between multifaceted lifestyle practices and the risk of migraine or TTH. By integrating diverse behavioral metrics, the study aims to capture the complex interplay among lifestyle factors, thereby overcoming the narrow scope of prior research and providing a more holistic understanding of how cumulative lifestyle patterns influence headache pathogenesis.

## 7. Limitations

Existing studies on the association between lifestyle behaviors and the risk of migraine or TTH exhibit notable inconsistencies, which can be attributed to multiple research limitations. First, heterogeneity in sample characteristics has influenced research outcomes. Studies have enrolled participants with broad age ranges and unbalanced geographical distributions, without adequately accounting for physiological variations across age groups and regional differences in medical resources and environmental exposures. For instance, the pathophysiological mechanisms of headache differ between adolescents and older adults, yet some studies omitted stratified analyses. Additionally, climatic factors and dietary habits in different regions may influence headache episodes, but most studies did not incorporate these variables into their analyses.

Second, disparities in research designs exacerbate conflicting conclusions. Although cross-sectional studies enable rapid acquisition of large datasets, they struggle to clarify the temporal sequence between lifestyle factors and headache onset, hindering valid causal inference. Cohort studies, while superior in establishing temporal sequence, suffer from issues such as inconsistent follow-up durations and high attrition rates, reducing the reliability and reproducibility of results. Moreover, inadequate control of confounding factors remains critical: many studies failed to fully adjust for potential confounders, such as the complex interactions among physical activity levels, sleep quality, and dietary patterns. Some studies also overlooked the impact of genetic predisposition on headache risk, making it difficult for results to reflect true associations accurately.

## 8. Research and clinical practice recommendations

The variability in research findings concerning the link between lifestyle behaviors and the risk of migraine and TTH is primarily due to methodological limitations related to sample characteristics, study designs, and confounding control. This highlights the critical need for mechanistic elucidation, longitudinal research, and evidence-based clinical strategies to fill existing knowledge gaps.

Study design and sample characteristics: Stratified Sampling: Implement stratified sampling based on age, region, and lifestyle to account for physiological differences (such as between adolescents and the elderly) and environmental factors (such as climatic zones, dietary habits); Longitudinal cohort studies: Conduct prospective cohort studies with standardized follow-up durations of at least 5 years to establish temporal causality between lifestyle behaviors and headache onset. Minimize attrition bias through structured participant retention strategies; and Multi-center collaboration: Engage in international or multi-regional collaborations to reduce geographical sampling bias and enhance the generalizability of the findings.

Mechanistic and pathophysiological research: Molecular mechanisms: Investigate neuroinflammatory markers (e.g., cytokines, chemokines) and pain regulatory pathways (e.g., the trigeminal-vascular system, descending analgesic pathways) to distinguish the underlying mechanisms between migraine and TTH; Genetic epidemiology: Conduct genome-wide association studies to identify susceptibility loci and gene-environment interactions,particularly in relation to lifestyle factors (e.g., sleep, stress); and Biomarker development: Validate objective biomarkers (e.g., cerebrospinal fluid markers, functional MRI patterns) for headache subtyping and prognosis.

Statistical methods and confounding control: Advanced modeling: Apply multivariate regression analysis with propensity score matching to adjust for complex confounders (e.g., physical activity, comorbid depression); Mediation analysis: Use structural equation modeling to elucidate mediating factors (e.g., hypothalamic-pituitary-adrenal axis dysfunction) in the association between lifestyle behaviors and headache; and Meta-analysis standardization: Conduct umbrella reviews with strict inclusion criteria to synthesize inconsistent findings across different study designs.

Furthermore, establishing registries that integrate longitudinal research data with electronic health records is essential for generating real-world evidence. Updating clinical practice guidelines based on emerging mechanistic insights (e.g., neuroinflammation-targeted therapies) and robust epidemiological evidence is also a crucial step for advancing translational research in this field.

## 9. Conclusion

Consistent with these findings, the present study concludes that no significant association was observed between lifestyle behaviors (alcohol consumption, BMI, smoking, physical activity) and the risk of migraine or TTH. Further studies are warranted to explore this association. However, only 7 studies were included in this analysis, so the conclusion requires further verification through additional high-quality research.

## Author contributions

**Data curation:** Xiaxia Jin.

**Funding acquisition:** Tao Gao.

**Investigation:** Xiyan Zhang, Xiaxia Jin.

**Methodology:** Tao Gao, Hongdan Luo, Xiyan Zhang.

**Software:** Hongdan Luo, Xiaxia Jin.

**Supervision:** Zhengquan Lei.

**Writing – original draft:** Tao Gao.
